# The Dissimilar Impact in Atrial Substrate Modificationof Left and Right Pulmonary Veins Isolation after Catheter Ablation of Paroxysmal Atrial Fibrillation

**DOI:** 10.3390/jpm12030462

**Published:** 2022-03-14

**Authors:** Aikaterini Vraka, Vicente Bertomeu-González, Lorenzo Fácila, José Moreno-Arribas, Raúl Alcaraz, José J. Rieta

**Affiliations:** 1 BioMIT.org, Electronic Engineering Department, Universitat Politecnica de Valencia, 46022 Valencia, Spain; aivra@upv.es; 2 Cardiology Department, Saint John’s University Hospital, 03550 Alicante, Spain; vbertog@gmail.com (V.B.-G.); jomoreno@gmail.com (J.M.-A.); 3 Cardiology Department, General University Hospital Consortium of Valencia, 46014 Valencia, Spain; lfacila@gmail.com; 4 Research Group in Electronic, Biomedical and Telecommunication Engineering, University of Castilla-La Mancha, 16071 Cuenca, Spain; raul.alcaraz@uclm.es

**Keywords:** atrial fibrillation, catheter ablation, coronary sinus, catheter channels, P-waves, local activation waves, left pulmonary veins, heart rate variability

## Abstract

Since the discovery of pulmonary veins (PVs) as foci of atrial fibrillation (AF), the commonest cardiac arrhythmia, investigation revolves around PVs catheter ablation (CA) results. Notwithstanding, CA process itself is rather neglected. We aim to decompose crucial CA steps: coronary sinus (CS) catheterization and the impact of left and right PVs isolation (LPVI, RPVI), separately. We recruited 40 paroxysmal AF patients undergoing first-time CA and obtained five-minute lead II and bipolar CS recordings during sinus rhythm (SR) before CA (**B**), after LPVI (**L**) and after RPVI (**R**). Among others, duration, amplitude and atrial-rate variability (ARV) were calculated for P-waves and CS local activation waves (LAWs). LAWs features were compared among CS channels for reliability analysis. P-waves and LAWs features were compared after each ablation step (**B**, **L**, **R**). CS channels: amplitude and area were different between distal/medial (p≤0.0014) and distal/mid-proximal channels (p≤0.0025). Medial and distal showed the most and least coherent values, respectively. Correlation was higher in proximal (≥93%) than distal (≤91%) areas. P-waves: duration was significantly shortened after LPVI (after **L**: p=0.0012, −13.30%). LAWs: insignificant variations. ARV modification was more prominent in LAWs (**L**: >+73.12%, p≤0.0480, **R**: <−33.94%, p≤0.0642). Medial/mid-proximal channels are recommended during SR. CS LAWs are not significantly affected by CA but they describe more precisely CA-induced ARV modifications. LPVI provokes the highest impact in paroxysmal AF CA, significantly modifying P-wave duration.

## 1. Introduction

Atrial fibrillation (AF) is the prevailing cardiac arrhythmia in the western world. Prolonged lifespan and the connection with a plenty of other comorbidities contribute to the ever-growing AF incidence. Health and economic burden caused by AF alert the need for thorough investigation on its pathophysiology [1]. AF springs principally from pulmonary veins (PVs) [2] and propagates through cardiac structures [3]. The main mechanism assisting the AF propagation is structural remodeling and fibrosis is especially contributing to the alteration of the cardiac anatomy, causing conduction heterogeneity, hence favoring the AF perpetuation [1,4]. Although conduction heterogeneity is more prominent during AF, the anatomical substrate can still be present for both atria even when patients are in sinus rhythm (SR) [5,6,7]. As PVs are the main AF foci, their electrical isolation, called catheter ablation (CA), is the star AF treatment [1,8]. Despite the high CA success rates for paroxysmal AF patients, persistent AF cases often require the CA of additional cardiac structures that trigger or propagate the AF activity, known as non-PV triggers [1,3,4,9,10,11,12].

Many techniques exist to localize non-PV triggers, with complex fractionated atrial electrograms (CFAEs) during AF [4,13,14] or low voltage electrograms (EGMs) during SR [4,15,16] being two of the most established ones. A combination of both techniques along with highly proportioned EGM fractionation has recently indicated sites showing fibrosis, with a high correlation between these areas in AF and SR [6]. Nevertheless, the effect of CA on additional non-PV triggers remains quite controversial. Evidence shows that additional ablation of these sites offers little or no improved results with respect to single PVs ablation [17,18,19]. It remains unclear, however, whether failure of additional CA applications to provide significant improvement in termination of AF stems from the incapacity of CA on sites other than PVs to terminate AF or from a vague and unclear definition of areas in need of ablation due to highly complex EGMs, thus highlighting the need for more reliable algorithms able to properly evaluate the atrial substrate [6,14].

So far, CA outcome on paroxysmal AF patients is primarily assessed from the analysis of the characteristics of P-waves, which represent the activation of the atria or heart-rate (HR) variability (HRV) analysis, which assesses the ventricular response, controlled by the autonomous nervous system (ANS). P-wave duration (PWD) is the most popular P-wave feature, reflecting the overall time that the wavefront needs to be propagated throughout the atria. Existence of prolonged or short PWD is considered an indicator of AF recurrence in paroxysmal or persistent AF patients, caused by conduction heterogeneity and scarring or shortening of the atrial refractory period, respectively [20,21,22,23,24,25]. PWD shortening is connected with the elimination of the conduction heterogeneity, hence being a favorable CA marker, while it is the second P-wave part, corresponding to left atrial depolarization, that is mainly modified after CA, possibly due to vicinity with PVs, the main object of CA [26,27,28,29]. PWD analysis goes beyond CA procedures, with application in studies predicting the AF occurrence or the risk for higher AF burden after pacemaker implantation [25,30,31].

Apart from PWD, P-wave dispersion, amplitude, area or P-wave to R-peak interval are popular features utilized to predict AF recurrence [26,27,32,33]. P-wave analysis has been additionally applied to frequency domain in order to discern among healthy and AF subjects [34]. HRV is a marker of fine tuning of ANS, which consists of sympathetic and parasympathetic systems and controls sinus rhythm. Evidence shows that people with low HRV are susceptible to AF [35,36,37]. Energy delivered by radiofrequency (RF) CA (RFCA) can disturb the balance between sympathetic and parasympathetic systems, by stimulating the former and leading to temporary withdrawal of the latter, hence causing HRV attenuation, which in turn has been associated with AF recurrence [38,39,40].

The number of studies and techniques aiming to analyze the CA effect on the atrial substrate is endless. At the same time, critical CA steps and their impact on the atrial substrate alteration is a rather neglected analysis field. Firstly, the aforementioned studies observing P-wave and HRV alterations only employ recordings acquired before and after CA. This postulates the theory of a uniform impact of left (LPVI) and right PVs isolation (RPVI). It should be considered, though, the possibility of each PV side playing a different role in atrial substrate alteration and hence, in AF activity, a conjecture that can be easily verified by employing signals recorded in between the ablation of LPVI and RPVI, which are already available in the recordings of any electrophysiology laboratory during stepwise CA procedures.

Coronary sinus’ (CS) strategical position between left (LA) and right atrium (RA) allows the detection of non-PV triggers and PV reconnection gaps throughout the atria during CA procedures via CS catheterization [41,42,43,44,45,46,47,48,49]. Despite its extensive use as a CA reference, whether CS analysis could provide reliable information with respect to the AF substrate modification or which channels of CS catheter are the most appropriate for the analysis are two vital issues that remain unexplored. During CS cannulation, the most proximal pair of electrodes (9–10) is placed close to RA and the most distal pair (1–2) close to LA [44,47]. Notwithstanding, CS catheterization may be rather challenging due to variable CS anatomy and shape, aggrravated by myocardial contraction or the existence of CS dilation, factors that can lead to unstable recordings, especially from the distal tip of the catheter [50,51,52,53]. Additionally, anatomical alterations of the, adjacent to CS extremes, mitral annulus across the cardiac cycle in SR may affect furthermore the stability of recordings acquired from distal and proximal electrodes of the CS catheter [54,55]. Considering the aforementioned factors, information recorded across the CS catheter electrodes could vary significantly and the choice of the appropriate channel recruited for the analysis should be made with extreme caution.

The present work aims to elucidate the aforeposed issues regarding the CA procedure, in order to arise the understanding on the mechanisms of important CA steps and their interaction with the CA result. In the first place, the ability of CS channels to describe with the highest precision possible the AF dynamics during SR is assessed and the most and least recommended CS channels are defined. Afterwards, the relevance of CS in substrate modification evaluation due to CA is investigated via analysis of features traditionally applied to ECG recordings and cross-referenced by P-waves and HRV analysis of the ECGs. Finally, the evolution of P-waves and CS LAWs after isolation of either sides of PVs is tracked in order to define the PV side that has the highest impact in atrial substrate modification due to CA.

The manuscript is organized as follows. Section 2 briefly describes the database recruited for the analysis, as well as the preprocessing and analysis steps. Section 3 presents the results, which are further interpreted in Section 4. Main findings are stated in Section 5.

## 2. Materials and Methods

### 2.1. Database

Initial database consisted of 61 paroxysmal AF patients without any previous CA sessions. Twenty-one patients were discarded due to extremely low amplitude or presence of noise and artifacts in the extreme channels of the CS recordings, probably due to dynamical change of mitral annulus anatomy during SR and vigorous myocardial contraction causing the movement of the CS cathether. The final database consisted of the remaining 40 patients.

**Figure 1:**
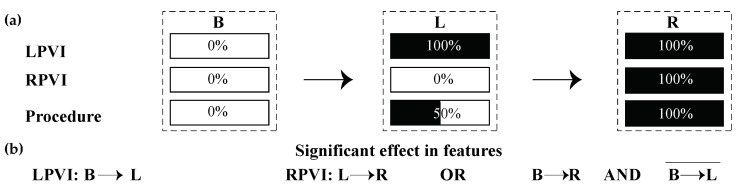
(**a**) Steps of CA procedure for which recordings were extracted and analyzed. In step **B**, no ablation has been performed yet (0%). In step **L**, LPVI has been completed (100%) and hence, we are in the middle of the procedure (50%). Step **R** corresponds to RPVI and to the end of the procedure. Therefore, each step is completed (100%). (**b**) Conditions in order for LPVI or RPVI to have a significant effect on the features under analysis. LPVI: left pulmonary vein isolation; RPVI: right pulmonary vein isolation.

Recordings from a standard 12-lead electrocardiogram (ECG) and a decapolar CS catheter with sampling frequency at 1 kHz were acquired by a Labsystem™ PRO EP recording system (Boston Scientific, Marlborough, MA, USA). Five-minute continuous segments before RFCA initiation (step **B**), after LPVI (step **L**) and after RPVI, which coincides with the end of the RFCA procedure ( step **R**), were chosen. The evolution of each ablation step is illustrated in Figure 1a. Step **B** corresponds to 0% of the procedure, step **L** corresponds to 100% of LPVI, 0% of RPVI and 50% of the total ablation procedure, while step **R** corresponds to 100% of the overall procedure. The effect of ablation of each PV side is shown in Figure 1b. A statistically significant difference in features between steps **B** and **L** indicates that LPVI is critical in atrial substrate modification, while a difference between steps **L** and **R** or between **B** and **R** but without the difference between steps **B** and **L** being statistically significant, proves a significant effect of RPVI. Surface analysis was limited to lead II, as P-waves are more prominent in this lead [56].

For the study of the reliability of CS channels in preserving the AF dynamics, final database consisted of a total of 58 step **B** or **R** recordings from the 40 patients of the database. CS catheter consisted of the following channels of bipolar signals: distal (D), mid-distal (MD), medial (M), mid-proximal (MP) and proximal (P), with D channel being the closest to LA and P channel to the RA. Firstly, a multichannel comparison allowed us to define the channels that can record the AF dynamics to the most reliable degree. Selection of the analysis channel for the CS study was then performed among the most robust channels, with unique favorable criteria the high signal amplitude and low baseline fluctuation. Specific attention was paid so that the same channel would be employed for all CA steps for the same patient.

Regarding the CA procedure, all patients underwent circumferential RFCA of PVs, guided by 3-D electroanatomical mapping during SR. RFCA was initiated by performing a crown surrounding left PVs (step **L**), followed by a crown surround right PVs (step **R**). Non-inducibility of AF was confirmed by pacing in all patients and was the endpoint of the procedure.

### 2.2. Preprocessing

For ECG recordings, powerline interference and high frequency muscle noise were removed by a wavelet-based denoising method [57] followed by a bidirectional low-pass filter with cut-off requency at 70 Hz [58]. Baseline wander was also removed [58].

EGM denoising and mean removal were the first preprocessing steps for CS recordings analysis [59]. Although presence of ventricular activity is not dominant in atrial bipolar signals, far-field activity in line with the R-peak of the ECG recordings has been observed in some cases. Removal of ventricular activity was performed with an adaptive cancellation method [60].

Next, ectopic beats correction was performed. Ectopic beats are premature atrial or ventricular contractions that affect the HRV. In our analysis, ectopic beats in ECGs, if present, did not exceed 4% of total beats. Their correction included the detection and cancellation of the premature complexes and their replacement by a new beat via linear interpolation [61]. Among various ectopic replacement methods, linear interpolation was chosen due to its better performance for time-domain HRV features [62].

Finally, detection and delineation of atrial activations was carried out. P-waves were firstly detected by an adaptive search window prior to the R-peak [63] and then delineated [64]. Local activation waves (LAWs) of CS were detected with an algorithm based on an alternative Botteron’s technique [65]. Delineation was performed by firstly smoothing the LAW with a five-point moving average filter [66]. Delineation of both ECG and intracardiac recordings was visually inspected and corrected, if needed, by an expert.

### 2.3. Main Analysis

**Figure 2:**
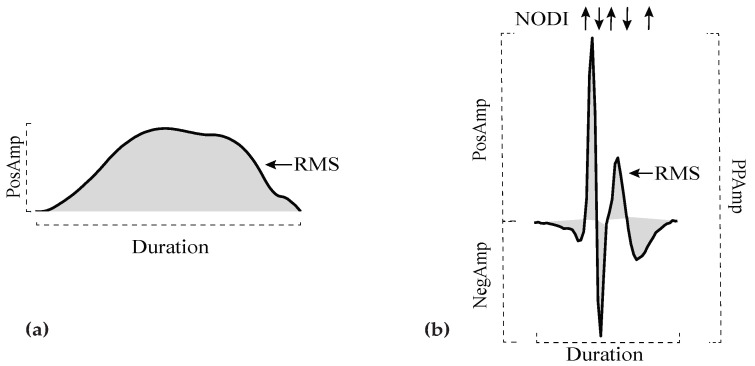
(**a**): Duration, amplitude, RMS and area (shaded) for a P-wave. (**b**): Duration, amplitude, RMS, area (shaded) and NODI for a LAW. Upward arrows represent an inflection, while downward arrows a deflection. In the figure, LAW has 3 major inflections and 2 major deflections. RMS: root mean square; NODI: number of deflections and inflections; LAWs: local activation waves.

Once preprocessed, the duration, amplitude, root mean square (RMS) value, area, number of deflections and inflections (NODI) and slope rate were calculated for P-waves and LAWs, as shown in Figure 2. Final values of these features were calculated by signal-averaging. A brief description of these characteristics is provided as follows. Further details are described elsewhere [66].

1. *Duration:* Distance between the onset and offset of each activation.
2. *Amplitude:* Amplitude of positive and negative maximum of each activation were considered as positive (*PosAmp*) and negative amplitude (*NegAmp*), respectively. Peak-to-peak amplitude (*PPAmp*) was the distance between positive and negative maximum points. As P-waves are positive in lead II, only maximum amplitude was calculated for ECG analysis.
3. *RMS:* Let $X_{n}$ be a time-series, so that $X_{n}=\left\{x_{1},\;x_{2},\ldots ,x_{n}\right\}$. *RMS* value is the quadratic mean of the function that defines the time-series. In our case, this function is defined by either the P-wave or LAW waveform.
4. *Area:* Area is calculated as the integration of the signal over the time interval. Trapezoidal method allows this integration, by splitting each signal into smaller and easier to calculate trapezoids. Final area is defined by the cumulative sum of these trapezoids. As LAWs contain both positive (*PosAr*) and negative (*NegAr*) parts, this method was separately applied to each one of them.
5. *NODI:* Deflections and inflections were calculated from the points that cross two auxiliary baselines, at ±25% of the signal amplitude. This metric was only calculated for LAWs, as P-waves do not show multiple major deflections and inflections.
6. *Slope rate:* The rhythm of increasing or decreasing slope was calculated at sample points equal to i% of the activation duration, with i=5, 10, 20. Slope rate at the maximum point was also computed. The equation calculating these slope rates was the following:
  (1) $$S_{i}=\frac{Amp\left(t_{i}\right)-Amp\left(t_{onset}\right)}{t_{i}-t_{onset}},$$
  where $Amp(t_{i})$ is the amplitude at the i% of the activation duration, $Amp(t_{onset})$ is the amplitude at the onset of the activation, $t_{i}$ is the sample point at the i% of the activation duration and $t_{onset}$ is the sample point corresponding to the onset of the activation.

Afterwards, features calculated across each recording were analyzed: morphology variability (MV), dispersion and time-domain HRV features.

1. *MV:* A reference signal was firstly created by the 20 most similar activations of the channel under analysis and then correlated with each and every activation, using an adaptive signed correlation index (ASCI) with 12% tolerance [67]. MV was then defined as the percentage of signals that correlated <95% with the reference signal.
2. *Dispersion:* Traditionally, for the calculation of dispersion, more than one ECG lead is employed and the difference between maximum and minimum activation duration across channels is computed. Alternatively, in our case, lead II was just extracted and P-wave dispersion analysis was performed in this channel because atrial activity presents the highest amplitude. Dispersion was then defined as the difference between the 25th and 75th percentiles of the atrial activations duration of each recording. This way, the effect of signal delineation accuracy is minimized and an extremely long or short activation caused by various factors will not affect significantly the results [68]. Dispersion of EGM recordings was calculated in the same way.
3. *Time-domain HRV features:* HRV analysis is normally performed on R-R intervals, thus describing ventricular response. As in the present work we are focused on atrial analysis, we modified the techniques by substituting R-R peaks by P-wave to P-wave for ECG and LAW to LAW for EGM recordings. As these features describe the atrial response, thus neglecting the effect of the atrioventricular node and other cardiac structures, they will be referred in the remainder of this document as atrial rate variability (ARV) features. Standard deviation of normal-to-normal beat interval (*SDNN*), variance of normal to normal beat interval (*VARNN*) and RMS of successive interbeat differences (*RMSSD*) were calculated for each recording.

#### Heart Rate Adjustment

Time-domain features of P-wave analysis are affected by variable HR [69]. More specifically, as HR increases, intervals between fiducial points of ECGs shorten. Therefore, HR adjustment is proposed in order to moderate this effect. For this purpose, additionally to the original analysis, a simple HR-adjustment factor is performed. As sampling frequency is 1 kHz, a 60 beat-per-minute recording would show one activation every 1000 sample points. However, as HR is diverse and often deviant from these values, the adjustment factor for the $i^{\mathrm{th}}$ activation was set as

(2) $$adj\left(i\right)=\frac{1000}{IBI_{i}},$$

where $IBI_{i}$ is the interbeat interval between the $i^{\mathrm{th}}$ and the ${\left(i-1\right)}^{\mathrm{th}}$ activations. Duration and area were normalized by this factor, while slope rates were inversely scaled by it. HR-adjusted values will be shown as HRA(y), where y=duration, area or $S_{i}$.

### 2.4. Statistical Analysis

Normality and homoscedasticity were tested with Saphiro-Wilk and Levene tests, respectively [70,71]. According to the results, non-parametric tests were employed for the comparison between populations.

Reliability analysis of the CS channels with respect to AF dynamics was performed on *Duration*, *Amplitude*, *RMS*, *Area* and *NODI*. In the first place, a multichannel comparison via a Kruskal-Wallis (KW) test was employed [72]. Comparison in pairs of two channels was performed with a Mann-Whitney U-test (MWU) [73] with Bonferroni correction. Median values were also calculated at each channel and any significant differences between each one and the remaining channels was explored by as well using a MWU with Bonferroni correction. Afterwards, a reference signal for each channel was calculated as described from MV analysis in Section 2.3. Then, the correlation between the morphology of each channel’s reference signal in pairs of two was calculated for each recording, using an ASCI with 12% tolerance.

For P-waves, LAWs and ARV analysis, comparison between ablation steps is performed with KW and post-hoc tests to define which step is crucial are performed with MWU with Bonferroni correction and median and interquartile range calculations. Analysis is performed for P-waves and CS LAWs separately. Percentage of variation (POV) of features was specified for each recording and between two ablation steps as

(3) $$POV\left(r_{i}\right)=\left(\frac{V_{2}}{V_{1}}-1\right)\times 100\left[\%\right],$$

where $r_{i}$ is the recording of the ith patient, $V_{2}$ is the value of each feature at the posterior step and $V_{1}$ is the value of the same feature at the prior step. How POV was modified between ablation steps **B-L** and **L-R** for both P-waves and LAWs was tested with MWU. Finally, HR was measured at each ablation step and compared among all steps with KW.

## 3. Results

### 3.1. Analysis of CS Features between Channels

Table 1 shows the results of the multichannel comparison as well as the comparison in pairs of channels, for the selected features. *Amplitude* and *Area* show different values among the CS catheter channels. Paired analysis showed that differences are mostly located between D and M or D and MP channels. A trend has also been observed between values of MD and M channels. Due to Bonferroni correction, α is 0.005, as 10 paired comparisons have been performed. Comparison between D-MD, MD-P and M-MP channels are not illustrated, as they did not show any statistically significant differences (p>0.03 , 0.38 and 0.35, respectively).

**Figure 3:**
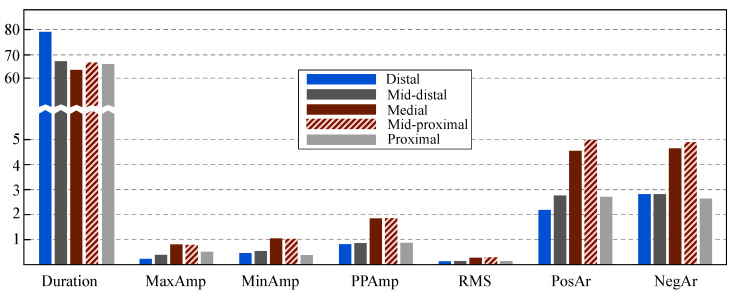
Bar graph for the median values of the analyzed features at each one of the CS channels. Note the break in the vertical scale for the feature *Duration*.

Figure 3 shows the median values obtained for each analyzed feature from the CS. As can be seen, the distal channel showed the longest LAWs *Duration* and lowest LAWs *Amplitude* and *Area* values for most of the features. Medial channel contained the shortest LAWs *Duration* and highest LAWs *Amplitude* values. *Area* was higher in mid-proximal channel, followed by medial channel. Mid-proximal channel showed overall similar behavior as medial channel, with rather high *Amplitude* and short LAWs *Duration* values.

The analysis of channels that varied significantly with respect to the others at each feature is shown in Table 2. Statistical comparison allows the detection of the channels that show the most and least deviant values and can corroborate the results shown in Figure 3.

None of the channels showed a statistically different LAWs *Duration* comparing to the remaining ones. Nevertheless, a trend was observed for distal and medial channels. Distal and medial channels showed additionally statistically different values with respect to the remaining channels regarding LAWs *Amplitude* and *Area* features. Mid-proximal channel also showed statistically significant difference than the other channels for positive *Amplitude* and a trend for the remaining *Amplitude* and *Area* features. Combining the aforementioned observations with the median values of the LAWs features for each CS channel presented in Figure 3, we can conclude that *Ampitude* and *Area* are statistically smaller in distal channel and higher in medial channel of the catheter, while duration tends to be longer in distal and shorter in medial channels. Mid-proximal channel shows a trend for high *Amplitude* and *Area* values as well.

**Figure 4:**
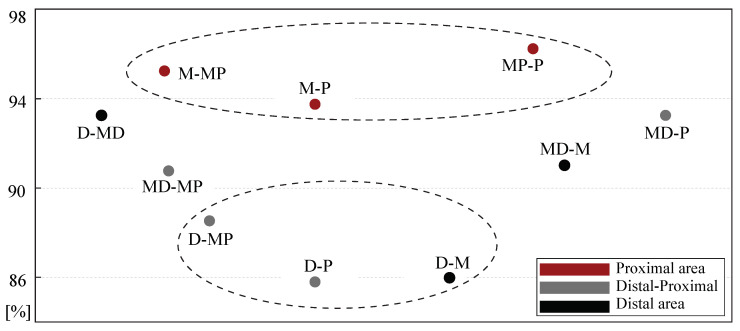
Correlations, as percentage, between the LAWs morphology in various CS catheter channels. The upper ellipse area contains the strongest and the bottom ellipse area the weakest correlations. Stronger correlations are found in proximal area, while moderate or weaker correlations are observed in distal area or between channels that are spatially far away (distal-proximal area).

Finally, how LAWs morphology of each channel correlated with the morphology of LAWs at each of the remaining channels can be appreciated from Figure 4. Channels of proximal area (MP, P) show higher correlations between their LAWs morphology compared to correlations from distal area (D, MD). Additionally, medial channel showed stronger correlation with proximal (93–95%) than distal area (86–91%). Although all adjacent channels showed relatively strong correlations, the highest values were observed in proximal area.

### 3.2. Analysis of Features from P-Waves and LAWs

The HR measurements did not reveal any statistical difference between HR at different ablation steps. However, a decrease in HR was observed in step **L**, as shown in Table 3.

Table 4 shows the median and interquartile range for the calculated features in P-waves at each ablation step. Multiple comparison among ablation steps is then shown in the fifth column (KW) and comparison of each feature between ablation steps in pairs of two using Bonferroni correction can be observed in the last three columns (MWU). *Duration* varied statistically among channels. When analysis in pairs was conducted, this variation was detected between steps **B-L** and **B-R**. As **L-R** comparison did not show any significant variation, the **B-R** significance is probably due to the **B-L** significance. Hence, step **L** is considered the critical step for the reduction in *Duration*, which falls from 120 ms in the beginning of the procedure to 104 ms, almost the final value observed after the end of CA of PVs.

In the same context, amplitude showed a downward trend after step **L**. Despite the key role of step **L** in the modification of *Duration*, when HR adjustment was performed, this step did not show any effect in HRA(Duration) and neither did step **R**. However, modification of HRA(Duration) showed a trend when values in the beginning and the end of the procedure were measured (**B-R** comparison), possibly due to a cumulative effect of CA in AF substrate which can be better appreciated quantitatively when isolation is totally performed. Although *Area* modification was not originally found to be statistically significant at each of the ablation steps, HR adjustment revealed for it a trend in **B-L** comparison, falling from 26.10 mV × ms to 19.40mV×ms. Finally, it can also be observed that measurements after step **L** showed, in a non-significant level, lower *Amplitude* and *Area* values and higher ARV, *MV* values and *Slope rate* values than steps **B** and **R**.

Regarding LAWs, comparison of each feature among all channels revealed a significant variation of *RMSSD*. Apart from this observation, none of the features varied statistically at any of the ablation steps. However, some trends have been observed. The respective results are shown in Table 5. LAWs showed a trend for shortening between steps **B-R**, as shown by both *Duration* and HRA(Duration) results. *MV* showed a trend for amplification after step **L** (**B-L** comparison) which was almost statistically significant. All ARV features employed in this study showed an increasing trend after step **L** (**B-L** comparison) and a decreasing trend after step **R** (**L-R** comparison). Note that threshold for MWU is α=0.0167 due to Bonferroni correction.

POV of all features between each two ablation steps are illustrated in Table 6 for both P-waves and LAWs. Comparison of POV that corresponds to successive step transitions **B-L** and **L-R** can also be seen in the last two columns. In general, POV in P-waves seem to be more prominent than respective POV values in LAWs, especially in the **B-L** comparison. *Duration* in P-waves got reduced by −13.30% after step **L**, while in LAWs by −5.49%. Step **R** did not additionally modify at average the P-waves at all (0.00% POV in **L-R** comparison). For LAWs, step R did reduce LAW *Duration* by an additional −1.93%. Comparison between **B-L** and **L-R** alterations in P-wave *Duration* presented a significant difference (p<0.0001), which was not observed in LAWs, indicating a different effect of LPVI and RPVI in P-wave but not in CS LAWs *Duration* alteration.

HR-adjustment did not have a different effect in **B-L** comparison of P-waves with respect to the same comparison for non-normalized *Duration* variation (−13.73% vs. −13.30%, respectively). However, HR-adjustment revealed a slightly higher, albeit not statistically significant, effect of step **R** in HRA(Duration), showing an incrementation of +1.60%. For LAWs, HRA(Duration) slightly mitigated the effect of step **L** (−3.89% for HR-adjustment) and potentiated the effect of step **R** (−4.46%). These results were not statistically significant either. *Amplitude* and HRA(PosAr) values showed the same kind of variations for P-waves and LAWs, with comparison between **B-L** and **L-R** transitions showing a trend for P-waves and low statistical power for LAWs.

**Figure 5:**
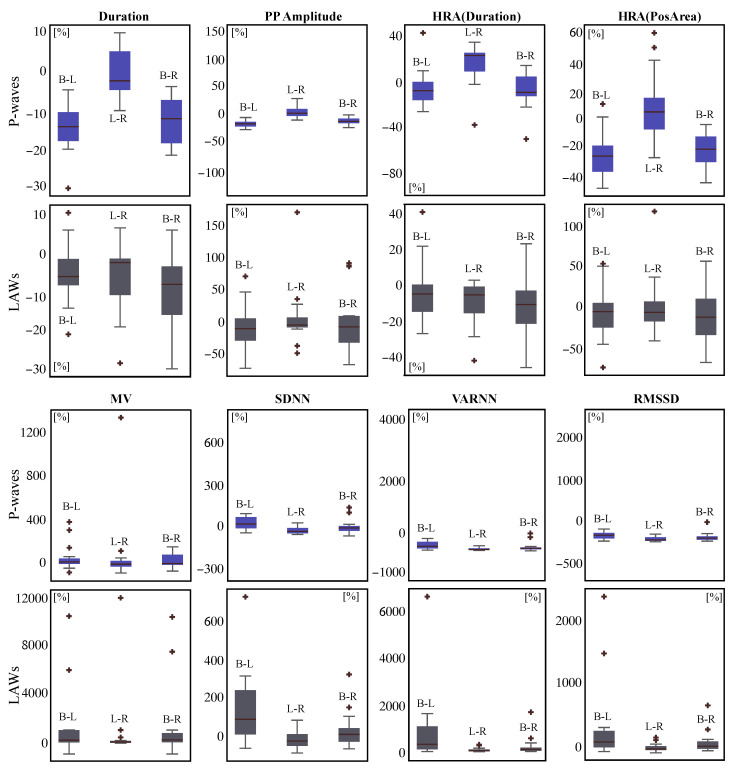
Representation of significant alterations illustrated as boxplots of POV for P-waves features (each **top**, blue) and LAWs features (each **bottom**, gray) between the defined ablation steps for selected features. Beware of the different vertical scale in each case for the same feature.

For the remaining features, *MV* showed a rather strong magnification after step **L** by +144.90% in LAWs, whereas the corresponding step in P-waves showed only a +15.00% of *MV* magnification. After step **R**, *MV* dropped by −5.93% in LAWs and by −6.11% in P-waves. Due to very intense variations in *MV* of LAWs, POV evolution between transitions from **B-L** and **L-R** varied significantly (p=0.0176). As already observed in Table 4 and Table 5, ARV features showed a notable incrementation after step **L** in both P-waves and LAWs. This is even more prominent in POV analysis, where ARV got increased by up to +225.90% in LAWs and up to +64.86% in P-waves (in *VARNN* in both cases). However, ARV after the end of the procedure (step **R**) got decreased by up to −55.91% in LAWs and −42.64% in P-waves (in *VARNN* in both cases). These very high variations in LAWs analysis led the **B-L** and **L-R** comparison to be statistically significant for all ARV features. As P-waves POV analysis also showed considerable variations but to a lesser extent, only POV in *RMSSD* varied statistically between **B-L** and **L-R**. Figure 5 then shows the POV box and whisker plots for the features that showed any kind of significant alteration or a trend, so that alterations in POV can be better illustrated. From y—axis can be additionally observed that POV in LAWs shows more scattered values that span along a wider range than P-waves analysis. This is noticeable at most of the boxplot pairs of Figure 5, but can be especially seen in the subplot of *MV*, where POV in P-waves is in the range of 0 to 1200% while in LAWs in the range of 0 to 12,000%.

## 4. Discussion

This multi-approach study had three main objectives. First of all, to define the CS channels that can record with the highest precision and robustness the AF dynamics during SR. The analysis revealed the existence of variability among CS channels especially in *Duration* and *Amplitude* features. Differences were mostly found betweeen distal and medial and between distal and mid-proximal, with a trend between mid-distal and medial channels. A combined interpretation of the analysis of medians and the one-vs-all analysis indicates that distal channel showed the longest *Duration*, whilst the shortest *Duration* has been recorded by the medial channel. Regarding *Amplitude* and *Area* values, these were smaller in distal channel and larger in medial and mid-proximal channels. Proximal area showed the strongest morphological correlations between its channels. On the contrary, correlations in distal area or between distal and proximal channels were weaker. Hence, being the least susceptible channels to exogenous factors during SR, medial and mid-proximal channels are recommended while distal and mid-distal channels are not suggested.

Various studies corroborate these conclusions. CS EGM fractionation analysis was related with AF recurrence during SR in proximal and medial but not in distal channel in a recent study [47]. Fractionation of proximal CS EGMs indicated AF patients in another study employing recordings during AF [74]. Another work found that AF cycle length analysis in distal channel failed to predict AF termination after CA. In the same study, only mid-proximal channel predicted freedom from AF recurrence [75].

The second objective of the present study was to investigate if CS recordings can describe adequately the substrate modification due to CA, as observed by P-wave analysis. Parallel P-waves and LAWs analysis has been conducted for this purpose. The former represent the entire atria while the latter provide very specific yet crucial information on CS function. Variations were observed to a higher degree in most of the features in P-waves than LAWs. *MV* and ARV features were modified to an exceptionally higher extent in LAWs than P-waves. As CS recordings are closer to the tissue under ablation than surface ECG recordings, variability caused by RF energy deliverance may be illustrated with higher precision by LAWs [38,39]. Variation was more consistent in P-waves than LAWs, while the latter showed higher *Dispersion* in values across all features.

The last but not least purpose of this work was the evaluation of additional recordings acquired during CA in order to understand the role that the ablation of each PV side plays to the modification of the studied features and, as a consequence, to the atrial substrate alteration. A significant P-wave shortening was observed after CA of both PV sides, in line with a plethora of previous studies mainly attributed to fibrotic areas causing conduction delays [20,21,22,23]. Interestingly enough, this reduction was observed right after LPVI, with RPVI not showing any additional effect to this feature. *Duration* before CA was 120 ms, dropping down to 104 ms after LPVI and showing a minor increase after RPVI, to 106.5 ms. P-wave *Amplitude* also tended to show a lower value after LPVI which was slightly increased after RPVI, but remained overall smaller than the pre-ablative measurements.

HRV attenuation after CA is considered an indicator of CA success [38,39,40]. In line with previous studies, ARV in the present study showed a non-significant reduction after the end of the procedure. Nevertheless, recordings obtained after LPVI showed a trend for amplification of ARV values by up to +64.86%. Previous works studying the effect of RF energy in rabbits and students in lying position found that RF exposure can cause HRV incrementation and HR attenuation [76,77]. These findings explain the aforementioned results of the present study. Additionally, HR was indeed found to decrease after LPVI in the present work. Finally, HR-adjustment preserved the variation that was observed in *Duration* after LPVI, although losing statistical power. It also incremented the different effect that RPVI had on *Duration*, showing a slight non-statistical increase of +1.60% after RPVI. The reason for this slight incrementation is the HR acceleration after the end of CA procedure with respect to recordings after LPVI, where RF energy deliverance was still going on. Higher HR leads to generally narrower P-waves, the size of which is retrieved after HR-adjustment. An additional factor that may have a minor effect on this incrementation is a possible deviation of 1–2 ms in P-wave delineation precision, which due to its size (<+2.00% of duration values range) is considered acceptable.

*Amplitude* in P-waves showed a trend for reduction after LPVI. Although final *Amplitude* was non-statistically reduced with respect to the beginning of the procedure, as in previous studies [27], RPVI slightly but non-significantly increased *Amplitude* values. Contrastly, *Slope rate* was non-significantly increased after LPVI but decreased after RPVI in most of the studied time instances. It is highly possible that RF exposure also has had an effect on *Amplitude* and *Slope rate* features, explaining these variations.

Regarding LAWs, variations of most features show weaker statistical power and lower POV at each ablation step with respect to P-waves analysis. As mentioned afore, *MV* and ARV varied more prominently in LAWs than P-waves. *MV* showed a high incrementation in the order of +144.9% after LPVI and a decrease of −5.93% after RPVI. The reasons for these dramatic changes are not clear. Exposure to RF energy, not only affecting ARV but also *MV* may be an explanation. Recently a study found *MV* in lead V1 P-waves of paroxysmal AF undergoing CA of PVs to decrease after the procedure, as a sign of a successful ablation [78]. Apart from the fact that these results come from P-waves analysis, which in our case show a slight attenuation overall, *MV* in the present analysis was extracted by a template of the 20 most similar activations and not by considering all activations of one recording. ARV was also increased after LPVI in LAWs and to a higher extent than P-waves (up to +225.9%), possibly due to proximity to the tissue under ablation as explained previously.

To our knowledge, this is the first complete comparative study to perform simultaneous P-waves and LAWs analysis on recordings obtained not only before and after but also during CA of PVs. Recently, a relevant study calculated the organization of ECG recordings before, during and after CA of PVs and did not find any step to affect significantly the organization indices under calculation [79]. As many differences exist with respect to our study, a comparison would not be straightforward. In the first place, individuals studied were persistent AF patients while the present study employed exclusively paroxysmal AF patients. As persistent AF shows more complicated atrial substrate and often presents AF drivers outside of PVs, efficiency of CA of PVs is notably lower with respect to success rates in paroxysmal AF patients [19,80]. Secondly, the procedure consisted of CA of PVs, CA of CFAEs and linear CA of LA. Recordings during the procedure were the recordings after CA of PVs and before CA of CFAEs and linear CA of LA. Hence, the intermediate stage of their analyis would be the final stage of ours and no information is provided about the contribution of left or right PVs. Finally, features employed are different from the features employed in the present study.

The key aspects of CA of PVs for paroxysmal AF patients investigated in the present study improve significantly the understanding of the AF mechanisms during SR and contribute to the knowledge on how these mechanisms respond to each step of CA. Moreover, the CA procedure itself is reconsidered and the most reliable means to analyze CS EGMs are explored. Overall, a more detailed perspective of the CA procedure and the effect of RF exposure to atrial tissue is obtained.

## 5. Conclusions

LPVI is the critical part of CA of PVs for paroxysmal AF patients, altering significantly the P-wave duration. RF exposure tends to cause temporary ARV incrementation, which is reversed right after the end of the CA procedure. The effect of CA of PVs on CS is less straightforward and takes place to a lesser extent. Thus, other atrial structures may be more indicative of the ablation outcome and should be assessed as alternative references.

It should be noted, however, that ARV modifications regarding RF energy are more prominently observed in CS LAWs, possibly due to the vicinity with the tissue under RF exposure. Hence, the employment of CS recordings may be beneficial for the study of ARV alterations during and after CA of PVs.

Finally, studies interested in employing CS analysis are encouraged to extract and investigate medial or mid-proximal channels, as they were found to be the most robust, showing the highest coherence between LAWs morphologies. Distal and mid-distal channels, on the other hand, should be avoided as they were prone to variable morphology and less clear activations.

## Figures and Tables

**Table 1:** Multichannel comparison (KW) and paired comparison (MWU) for the features defined in CS channels. Statistically significant results are shown with an asterisk (*). In MWU analysis, α=0.005. D: distal; MD: mid-distal; M: medial; MP: mid-proximal; P: proximal.

	KW	MWU						
		D-M	D-MP	D-P	MD-M	MD-MP	M-P	MP-P
*Duration*	0.2136	0.0188	0.0893	0.4355	0.3502	0.8927	0.2232	0.7001
*PosAmp*	<0.0001 *	<0.0001 *	<0.0001 *	0.0140	0.0053	0.0038 *	0.0355	0.0502
*NegAmp*	0.0005 *	0.0002 *	0.0016 *	0.4162	0.0087	0.0587	0.0080	0.0428
*PPAmp*	0.0001 *	<0.0001 *	0.0002 *	0.1476	0.0087	0.0182	0.0112	0.0491
*RMS*	0.0003 *	0.0001 *	0.0007 *	0.1644	0.0053	0.0409	0.0182	0.0950
*PosAr*	0.0008 *	0.0003 *	0.0020 *	0.1826	0.0080	0.0491	0.0182	0.0950
*NegAr*	0.0024 *	0.0014 *	0.0025 *	0.3384	0.0119	0.0347	0.0207	0.0758
*Deflections*	0.7925	0.6217	0.2458	0.3258	0.9749	0.5681	0.6126	0.7430
*Inflections*	0.8045	0.6324	0.7477	0.3711	0.9840	0.4349	0.7831	0.2354

**Table 2:** One-vs.-all analysis of defined features for each one of CS channels. Asterisks (*) indicate statistically significant values. Due to multiple comparison, α has been modified to 0.01.

Features	Distal	Mid-Distal	Medial	Mid-Proximal	Proximal
*Duration*	0.0472	0.8617	0.0837	0.6755	0.7345
*PosAmp*	<0.0001 *	0.1688	0.0021 *	0.0061 *	0.8176
*NegAmp*	0.0038 *	0.4720	0.0019 *	0.0446	0.1317
*PPAmp*	0.0008 *	0.2669	0.0017 *	0.0204	0.3261
*RMS*	0.0014 *	0.2762	0.0018 *	0.0394	0.3717
*PosAr*	0.0022 *	0.3799	0.0027 *	0.0610	0.3555
*NegAr*	0.0103	0.2427	0.0049 *	0.0526	0.3113
*Deflections*	0.3181	0.8899	0.8099	0.3745	0.6235
*Inflections*	0.6108	0.7069	0.7779	0.3422	0.4243

**Table 3:** Heart-rate at each ablation step and comparison between three steps (KW). As result indicated a non-significant comparison, no MWU has been performed. KW: Kruskal-Wallis; MWU: Mann-Whitney U-test; B: before CA; L: after LPVI; R: after RPVI.

	B	L	R
Median (iqr)	57.2 (17.0)	55.0 (12.0)	58.6 (13.4)
KW	0.7713		

**Table 4:** Median (interquartile) values for each feature and results for KW and MWU tests for P-waves. Statistically significant results are shown in (*). Due to Bonferroni correction, threshold for MWU (last three columns) is α=0.0167.

	Median			KW	MWU		
Features	B	L	R		B-L	B-R	L-R
*Duration* [ms]	120.0 (12.00)	104.0 (13.00)	106.5 (21.00)	0.003 *	0.001 *	0.009 *	0.558
*PosAmp* [mV]	0.428 (0.303)	0.354 (0.290)	0.374 (0.232)	0.084	0.055	0.097	0.319
*PPAmp* [mV]	0.431 (0.303)	0.356 (0.290)	0.374 (0.232)	0.084	0.056	0.097	0.319
*RMS* [mV]	0.263 (0.179)	0.214 (0.179)	0.230 (0.232)	0.144	0.103	0.150	0.275
*PosAr* [mV×ms]	24.63 (12.94)	16.57 (14.62)	20.39 (9.98)	0.141	0.103	0.103	0.438
$S_{5}$ [mV/ms]	0.005 (0.002)	0.007 (0.004)	0.006 (0.002)	0.162	0.110	0.235	0.211
$S_{10}$ [mV/ms]	0.007 (0.002)	0.008 (0.004)	0.007 (0.003)	0.178	0.117	0.420	0.150
$S_{20}$ [mV/ms]	0.010 (0.005)	0.011 (0.003)	0.009 (0.005)	0.336	0.384	0.693	0.133
$S_{max}$ [mV/ms]	0.010 (0.004)	0.009 (0.004)	0.008 (0.004)	0.823	0.987	0.602	0.602
HRA (Duration)	119.5 (57.39)	106.9 (26.04)	101.0 (36.91)	0.159	0.141	0.079	0.740
HRA (PosAr)	26.10 (16.94)	19.40 (14.16)	22.12 (14.01)	0.144	0.085	0.110	0.716
$\mathrm{HRA}\;(S_{5})$	0.004 (0.004)	0.006 (0.006)	0.006 (0.003)	0.367	0.261	0.235	0.537
$\mathrm{HRA}\;(S_{10})$	0.006 (0.003)	0.007 (0.007)	0.007 (0.004)	0.441	0.248	0.402	0.558
$\mathrm{HRA}\;(S_{20})$	0.010 (0.005)	0.010 (0.008)	0.009 (0.006)	0.801	0.693	0.837	0.517
$\mathrm{HRA}\;(S_{max})$	0.009 (0.007)	0.008 (0.006)	0.008 (0.006)	0.994	0.962	1.000	0.912
*MV*	0.605 (0.329)	0.753 (0.335)	0.675 (0.467)	0.476	0.189	0.624	0.646
*Dispersion* [ms]	12.00 (4.000)	11.00 (7.000)	10.00 (4.000)	0.310	0.208	0.176	0.949
*SDNN*	94.25 (55.32)	99.91 (71.96)	84.28 (62.10)	0.136	0.133	0.862	0.060
*VARNN*	$8.8\times 10^{3}$	$9.9\times 10^{3}$	$7.1\times 10^{3}$	0.136	0.133	0.862	0.060
	$\left(1.07\times 10^{4}\right)$	$\left(1.77\times 10^{4}\right)$	$\left(1.21\times 10^{4}\right)$				
*RMSSD*	95.44 (59.68)	126.79 (95.29)	92.51 (61.73)	0.136	0.052	0.962	0.069

**Table 5:** Median (interquartile) values for each feature and results for KW and MWU tests for CS LAWs. Statistically significant results are shown in (*). Due to Bonferroni correction, threshold for MWU (last three columns) is α=0.0167. As highest peak is often found in negative amplitude in LAWs, slope rate in $\mathrm{HRA}\;(S_{max})$ is negative.

	Median			KW	MWU		
Features	B	L	R		B-L	B-R	L-R
*Duration* [ms]	100.5 (14.00)	97.50 (18.00)	90.00 (23.00)	0.108	0.241	0.055	0.217
*PosAmp* [mV]	0.492 (0.987)	0.509 (0.893)	0.641 (0.775)	0.835	0.646	0.887	0.602
*NegAmp* [mV]	−0.831 (1.572)	−0.779 (0.772)	−0.915 (0.486)	0.892	0.740	0.912	0.646
*PPAmp* [mV]	1.361 (2.624)	1.382 (1.495)	1.570 (1.462)	0.942	0.740	0.937	0.837
*RMS* [mV]	0.150 (0.345)	0.151 (0.195)	0.181 (0.218)	0.847	0.740	0.912	0.558
*PosAr* [mV×ms]	4.407 (5.175)	3.718 (5.160)	3.985 (4.836)	0.896	0.670	0.837	0.788
*NegAr* [mV×ms]	4.085 (5.485)	3.818 (4.045)	3.992 (4.655)	0.864	0.624	0.937	0.693
*Deflections*	3.000 (1.000)	3.000 (1.000)	3.000 (1.000)	0.916	0.682	0.889	0.828
*Inflections*	3.000 (1.000)	3.000 (1.000)	2.500 (1.000)	0.915	0.878	0.810	0.695
$S_{5}$ [mV/ms]	$3.2\times 10^{-4}$	$3.9\times 10^{-4}$	$3.8\times 10^{-4}$	0.732	0.624	0.837	0.438
	$\left(2.1\times 10^{-4}\right)$	$\left(3.4\times 10^{-4}\right)$	$\left(5.0\times 10^{-4}\right)$				
$S_{10}$ [mV/ms]	$4.3\times 10^{-4})$	$4.4\times 10^{-4})$	$4.9\times 10^{-4}$	0.767	0.764	0.558	0.558
	$\left(6.0\times 10^{-4}\right)$	$\left(5.2\times 10^{-4}\right)$	$(4.8\times 10^{-4}$				
$S_{20}$ [mV/ms]	$3.8\times 10^{-4}$	$4.7\times 10^{-4}$	$5.3\times 10^{-4}$	0.932	0.887	0.962	0.669
	(0.003)	(0.001)	(0.001)				
$S_{max}$ [mV/ms]	−0.018 (0.058)	−0.019 (0.027)	−0.021 (0.044)	0.996	0.937	0.987	0.987
HRA (Duration)	104.8 (32.83)	102.1 (23.50)	91.25 (33.19)	0.159	0.669	0.060	0.200
HRA (PosAr)	4.446 (6.861)	3.503 (4.345)	3.832 (4.737)	0.882	0.669	0.693	0.937
$\mathrm{HRA}\;(S_{5})$	$3.6\times 10^{-4}$	$4.1\times 10^{-4}$	$3.4\times 10^{-4}$	0.753	0.558	0.837	0.517
	$\left(1.9\times 10^{-4}\right)$	$\left(3.9\times 10^{-4}\right)$	$\left(4.1\times 10^{-4}\right)$				
$\mathrm{HRA}\;(S_{10})$	$4.4\times 10^{-4}$	$4.2\times 10^{-4}$	$4.7\times 10^{-4}$	0.771	0.912	0.558	0.537
	(0.001)	$\left(3.6\times 10^{-4}\right)$	(0.001)				
$\mathrm{HRA}\;(S_{20})$	$4.1\times 10^{-4}$	$3.9\times 10^{-4}$	$5.1\times 10^{-4}$	0.836	0.710	0.812	0.580
	(0.003)	(0.001)	(0.001)				
$\mathrm{HRA}\;(S_{max})$	−0.015 (0.049)	−0.016 (0.030)	−0.019 (0.047)	0.967	0.887	0.788	0.912
*MV*	0.028 (0.048)	0.100 (0.103)	0.067 (0.351)	0.056	0.018	0.113	0.692
*Dispersion* [ms]	2.500 (3.000)	2.000 (4.000)	2.000 (6.000)	0.676	0.461	0.923	0.451
*SDNN*	74.18 (57.56)	96.51 (94.74)	61.75 (73.48)	0.056	0.048	0.912	0.041
*VARNN*	$5.5\times 10^{3}$	$9.4\times 10^{3}$	$3.4\times 10^{3}$	0.056	0.048	0.912	0.041
	$\left(7.9\times 10^{3}\right)$	$\left(2.0\times 10^{4}\right)$	$\left(1.1\times 10^{4}\right)$				
*RMSSD*	98.87 (86.99)	127.4 (119.8)	90.40 (74.41)	0.049 *	0.026	0.764	0.064

**Table 6:** POV for between every two ablation steps for P-waves and LAWs and comparison between POV of successive step transitions **B-L** and **L-R**. Statistically significant results are shown in (*). POV: percentage of variation.

	B-L [%]		L-R [%]		B-R [%]		MWU (BL-LR)	
Features	P-Waves	LAWs	P-Waves	LAWs	P-Waves	LAWs	P-Waves	LAWs
*Duration*	−13.30	−5.49	0.00	−1.93	−11.01	−7.46	< 0.0001	0.6576
*PosAmp*	−16.65	−3.58	3.86	0.52	−11.25	−6.51	0.0556	0.6464
*PPAmp*	−16.04	−7.79	3.80	−2.15	−11.52	−5.09	0.0556	0.7397
*RMS*	−18.51	0.82	7.51	19.91	−12.39	20.89	0.1032	0.7397
*PosAr*	−25.68	−5.23	6.50	−1.74	−20.72	−4.06	0.1032	0.6693
$S_{5}$	30.95	22.47	−13.75	−1.82	12.95	20.24	0.1101	0.6239
$S_{10}$	15.75	1.34	−11.23	11.74	−0.73	13.24	0.1173	0.7637
$S_{20}$	12.84	25.89	−18.35	12.20	−7.87	41.24	0.3843	0.8868
$S_{max}$	−5.40	4.37	−6.96	7.75	−11.99	12.46	0.9874	0.9370
HRA(Duration)	−13.73	−3.89	1.60	−4.46	−14.49	−9.84	0.1412	0.6693
HRA(PosAr)	−24.54	−2.90	6.60	−3.86	−19.65	−19.94	0.0847	0.6693
$\mathrm{HRA}(S_{5})$	58.69	14.18	−9.34	−16.49	43.87	−4.65	0.2614	0.5583
$\mathrm{HRA}(S_{10})$	13.41	−4.10	−4.38	11.80	8.45	7.21	0.2482	0.9118
$\mathrm{HRA}(S_{20})$	3.35	−5.64	−6.44	32.58	−3.30	25.10	0.6925	0.7160
$\mathrm{HRA}(S_{max})$	−6.60	4.11	−1.21	17.70	−7.74	22.53	0.9621	0.8868
*MV*	15.00	144.9	−6.11	−5.93	−0.42	172.1	0.1892	0.0176 *
*Dispersion*	−22.42	0.00	22.22	80.00	−9.55	0.00	0.2084	0.4613
*SDNN*	28.39	79.80	−24.27	−33.94	0.28	0.92	0.1249	0.0480 *
*VARNN*	64.86	225.9	−42.64	−55.91	0.57	1.93	0.1249	0.0480 *
*RMSSD*	45.15	73.12	−28.30	−36.30	0.26	5.43	0.0445 *	0.0257 *

## Data Availability

The data supporting reported results and presented in this study are available on request from the corresponding author.
